# 64. Hypertrophic pachymeningitis-myeloperoxidase antineutrophil cytoplasmic antibody-associated vasculitis or human immunodeficiency virus-related disease?

**DOI:** 10.1093/rap/rky034.027

**Published:** 2018-09-20

**Authors:** Priyanka Agrawal, Sunil Melath, Henry Penn

**Affiliations:** 1 Rheumatology, Northwick Park Hospital, Harrow, UNITED KINGDOM; 2 Rheumatology, Frimley Park Hospital, Frimley, UNITED KINGDOM

**Introduction:** Hypertrophic pachymeningitis is a rare clinical disorder involving localised or diffuse thickening of the dura mater mostly around the brain or rarely, the spinal cord. Most patients with hypertrophic pachymeningitis have chronic headaches with or without neurological manifestations such as cranial neuropathies and cerebellar ataxia. Hypertrophic pachymeningitis is thought to be associated with several granulomatous disorders: autoimmune diseases such as antineutrophil cytoplasmic antibody (ANCA) associated vasculitis, rheumatoid arthritis, sarcoidosis, and IgG4-related disorder; infections such as tuberculosis, syphilis, bacterial or fungal diseases; neoplasms such as lymphoma; and the idiopathic variety when evaluation fails to reveal a cause. This case report describes ANCA-associated vasculitis manifesting as hypertrophic pachymeningitis in a 68 year old male known to have chronic stable human immunodeficiency virus (HIV). A review of the literature did not reveal any other case of hypertrophic pachymeningitis in a patient with both retroviral disease and a positive ANCA.

**Case description:** A 68 year old male with chronic stable retroviral disease presented with bilateral profound hearing loss, progressive headaches and personality change. He gave a one year history of hearing loss in both ears following bilateral grommet insertion. He had associated tinnitus and unsteadiness. He gave a six week history of severe headaches that woke him up at night. He also reported a change in his personality with emotional lability. He had lost his appetite and had significant weight loss. He did not have any fever, haemoptysis, chest pain or shortness of breath. There was no history of haematuria. He was diagnosed with HIV thirteen years prior to this presentation. His past medical history also included ischaemic heart disease with a triple coronary artery bypass graft, hypertension and cerebrovascular disease. He had previously been treated for pneumocystis jiroveci pneumonia. He was compliant with anti-retroviral treatment. He stopped smoking twenty six years ago and did not drink alcohol. On examination, he was profoundly deaf. Glasgow Coma Scale was 15/15. Visual acuity was 6/6 on the right and 6/9 on the left. He had normal fundi. He had mild postural and action tremor with upper limb ataxia. He was unable to heel toe walk. There was no neck stiffness or photophobia. There was an unsustained horizontal gaze evoked nystagmus with jerky pursuit. Reflexes were brisk but plantars were flexor. There was a mild loss of vibration sense in his feet but sensation was otherwise normal. He had no skin rashes or synovitis in any joint. His chest was clear to auscultate and heart sounds were normal. Abdominal examination was normal. Blood tests showed erythrocyte sedimentation rate (ESR) 126 mm/hr, C reactive protein (CRP) 135 mg/L, HIV viral load <50 copies/ml, CD4 count 567 x 106/L, haemoglobin 114 g/L, white cell count 9.4 x 109/L, platelets 587 x 109/L. He had normal liver, kidney, lipid and bone profiles. Rheumatoid factor, antinuclear antibodies, extractable nuclear antibodies and syphilis serology were negative. He had a positive ANCA with myeloperoxidase (MPO) antibodies 155 IU/ml and proteinase 3 (PR3) antibodies 1.5 IU/ml. Immunoglobulin G level was 14.39 g/L with IgA 5.67 g/L and IgM 0.32 g/L. Complements were normal. Chest x-ray showed clear lung fields. MRA head with gadolinium showed diffuse marked smooth dural thickening with some subdural fluid collection, established right frontal infarct, right internal carotid artery stenosis in the carotid canal, and left sided middle ear cleft effusion. He had a lumbar puncture and cerebrospinal fluid examination showed an elevated protein of 1.03 g/L with normal glucose and lactate, and presence of oligoclonal bands (absent in serum). There were no organisms or malignant cells, and no HIV-1 RNA. JC virus PCR was also negative. He was diagnosed with hypertrophic pachymeningitis related to ANCA associated vasculitis. The consensus was that this is not a case of HIV-related disease. He was commenced on prednisolone 60mg daily. His headache responded dramatically to high dose steroid. However, his sensorineural hearing loss and unsteadiness persisted. Subsequently, the prednisolone dose was reduced and he was commenced on mycophenolate mofetil. Almost one year after his initial presentation, his headache has completely resolved, MPO ANCA has reduced to 44 IU/ml, CRP to 20 mg/L and ESR to 52 mm/hr. A repeat MRI head with gadolinium shows ongoing or recurrent pachymeningitis with no evidence of recent or interval parenchymal abnormalities and resolution of previously demonstrated subdural fluid collections. He remains on mycophenolate mofetil 1g twice daily and a reducing regimen of prednisolone (currently 10mg daily). He is profoundly ataxic and has been commenced on levetiracetam for worsening intention tremor.

**Discussion:** Hypertrophic pachymeningitis (HP) is an extremely rare disease caused by fibrosing inflammatory processes. It may occur as the first involvement in approximately half of patients with ANCA-associated vasculitis-related HP who frequently present with headache and/or cranial neuropathy as a common symptom, in addition to ENT involvement. Recent reports on patients with MPO ANCA positive HP indicate a relationship between HP and the limited form of granulomatosis with polyangiitis (GPA). In a previous study, 82% of MPO-ANCA-positive HP patients were diagnosed with GPA according to Watts' algorithm. Both seropositivity for MPO-ANCA and one surrogate marker for GPA (chronic sinusitis, otitis media, or mastoiditis for >3 months) were required for classification as GPA in patients with MPO-ANCA-positive HP. Several studies have reported the occurrence of ANCA in HIV-positive patients, ranging from 13% to 42%, with very few associated with clinical small vessel vasculitis. It has been proposed that ANCA in these patients is secondary to a marked polyclonal B-cell response to HIV infection. While autoantibodies may represent false positives in the context of active HIV with polyclonal gammopathy, true systemic autoimmune disease should be considered in patients with HIV infection presenting with consistent clinical features. Thus, as we increasingly find patients with reconstituted immune systems, due to effective antiretroviral therapy, autoimmune phenomena may be more common. HIV-associated pachymeningitis has not been well described, but CNS escape - the phenomenon of virologic suppression in plasma but persistent, detectable HIV RNA in CSF - has been described. In this case report, the co-existence and simultaneous improvement of pachymeningitis and ANCA titre indicated that pachymeningitis was a manifestation of ANCA-associated vasculitis. If left untreated, HP frequently progresses, and although it is initially steroid-responsive, clinical manifestations frequently flare up after a reduction in the dose of steroids, necessitating the addition of immunosuppressive agents.

**Key Learning Points:** Headache and cranial neuropathies are the most common clinical presentation of hypertrophic pachymeningitis. Our patient has a diagnosis of limited GPA with a positive MPO ANCA, as he had a history of otitis media preceding his presentation (Watts’ algorithm). True ANCA-associated vasculitis is rare in HIV but does occur and should be considered if the clinical findings support it.

**Disclosure: P. Agrawal:** None. **S. Melath:** None. **H. Penn:** None.

